# Trends in the prevalence of Chronic Kidney Disease in the United States, 1999–2018

**DOI:** 10.3389/fmed.2024.1499225

**Published:** 2025-01-15

**Authors:** Mansi Zhuang, Xiaogang Lv, Yanan Zhu, Nan Zheng, Yiqiang Zhan

**Affiliations:** ^1^Department of Epidemiology, School of Public Health (Shenzhen), Sun Yat-sen University, Shenzhen, China; ^2^Department of Health Education, Futian Institute of Health Education, Shenzhen, China; ^3^Institute of Environmental Medicine, Karolinska Institutet, Stockholm, Sweden

**Keywords:** Chronic Kidney Disease, prevalence, NHANES, temporal trends, United States

## Abstract

**Background:**

Chronic Kidney Disease (CKD) is an escalating public health concern in the United States, linked with significant morbidity, mortality, and healthcare costs. Despite known risk factors like age, hypertension, and diabetes, comprehensive studies examining temporal trends in CKD prevalence are scarce. This study aims to analyze these trends using data from the National Health and Nutrition Examination Survey (NHANES).

**Methods:**

This cross-sectional study analyzed NHANES data spanning 20 years (1999–2018), including 55,081 adults aged 20 years and above. Key renal function indicators like estimated glomerular filtration rate (eGFR) and albumin-to-creatinine ratio (ACR) were assessed, and CKD stages were categorized accordingly.

**Results:**

A fluctuating trend in CKD prevalence was observed, with early CKD stages (G1–G3) increasing from 9.28% in 1999–2000 to 12.93% in 2017–2018. Advanced CKD stages (G4–G5) showed a slight increase in prevalence from 0.3% in 1999–2000 to 0.51% in 2017–2018. Notable disparities were observed across age groups, diabetes status, and racial categories. Relatively, the elderly, women, and non-Hispanic whites have a higher prevalence of CKD, while individuals with diabetes have a consistently higher prevalence of early CKD from 1999 to 2018. The increasing prevalence of diabetes during the study period highlights its significant role as a CKD risk factor.

**Conclusion:**

The prevalence of CKD in the U.S. has been rising over the period 1999–2018, and varying across demographic groups, underscoring significant disparities and risk factors. These insights are crucial for healthcare planning, policy formulation, and targeted interventions for CKD management.

## 1 Introduction

Chronic Kidney Disease (CKD) presents a formidable challenge to public health, significantly influencing morbidity and mortality worldwide. The National Health and Nutrition Examination Survey (NHANES) has been instrumental in shedding light on the epidemiology of CKD within the U.S., providing invaluable insights (1). Notably, the prevalence of CKD varies considerably across different racial and ethnic groups, with marked disparities (2). Additionally, CKD is associated with a range of comorbidities, including metabolic-associated fatty liver disease (MAFLD) (3), severe mental illness (4), and COVID-19 (5). CKD risk factors encompass age, hypertension, diabetes mellitus, and hyperuricemia (6). Comprehending these factors is crucial for timely intervention and effective management. Despite this, studies are scarce to analyze trends in CKD prevalence using NHANES data, particularly those employing cross-sectional designs (7).

CKD research has seen remarkable developments in our comprehension of this intricate health issue. Initially, research primarily concentrated on ESRD and its direct causes. However, recent decades have witnessed a paradigm shift toward understanding the early stages of CKD and its wider epidemiological implications. In previous studies, pivotal studies by researchers such as James et al. (8) and González-Albarránet al. (9) began to unravel the multifaceted nature of CKD, identifying key risk factors like hypertension, diabetes mellitus, and obesity. These studies underscored the criticality of early detection and management of CKD, particularly given its often asymptomatic nature in the initial stages.

The role of NHANES in CKD epidemiology has been indispensable. As a continuous survey, it has provided longitudinal data, allowing researchers to track changes in CKD prevalence over time. For instance, a landmark study by Rao et al. (10) utilized NHANES data to demonstrate the increasing prevalence of CKD in the United States, linking it to the rising rates of diabetes and hypertension. NHANES has played an irreplaceable role in the epidemiology of CKD. A notable study by Hsu and Powe (11) utilized NHANES data to illustrate the escalating prevalence of CKD in the United States, correlating it with the increasing incidences of diabetes and hypertension.

Our current understanding of CKD has also been significantly enhanced by advancements in diagnostic criteria and methods. The adoption of the CKD-EPI equation (12) for estimating glomerular filtration rate (eGFR) marked a substantial improvement in accurately identifying stages of CKD. Additionally, the incorporation of albuminuria in CKD staging, as recommended by KDIGO guidelines (12), has improved the sensitivity of CKD detection. This evolving field underscores the necessity for continuous research to refine our understanding of CKD epidemiology.

In light of the rising prevalence of CKD and its associated risk factors, there is an imperative need to update our knowledge of current trends in CKD prevalence in the United States. This study aims to bridge this gap by conducting a comprehensive cross-sectional analysis of CKD prevalence trends in the United States, utilizing the NHANES database.

## 2 Methods

### 2.1 Study population

In our research, we leveraged data from NHANES (13), a comprehensive program designed to evaluate the health and nutritional status of adults and children in the United States. This cross-sectional study meticulously analyzed NHANES data collected over a 20 years, from 1999 to 2018. The dataset encompassed a diverse cohort of 55,081 adults aged 20 years and older. The NHANES employs a sophisticated, multistage probability sampling design to select a representative sample of the civilian, non-institutionalized U.S. population. This selection process involves a stratified method of choosing counties, blocks within these counties, households within these blocks, and ultimately, individuals within these households. This meticulous approach ensures proportional representation of various demographic groups, including different ages, races, and ethnicities, in the survey.

Data collection in this study was extensive, encompassing detailed demographic information, comprehensive health interviews, and thorough health examinations. Participants in the NHANES were subjected to an interview conducted in their homes, followed by an exhaustive physical examination at a mobile examination center (MEC). These examinations included a variety of health tests. Critical renal function indicators, such as serum creatinine and the albumin-to-creatinine ratio (ACR), were meticulously analyzed from the laboratory data collected during these examinations.

Moreover, the NHANES methodology includes a strategic oversampling of certain population subgroups that are more susceptible to health issues. These groups include older adults, African Americans, and Hispanics. This approach ensures the collection of ample data to reliably estimate health status indicators for these groups. In our study, we incorporated the NHANES-provided sampling weights in our analysis. These weights are designed to account for the complex survey design, non-response biases, and post-stratification adjustments, enabling us to produce results that accurately reflect the U.S. population.

### 2.2 CKD stages

Key renal function indicators including serum creatinine and ACR were analyzed. The eGFR was calculated using the CKD-EPI equation, primarily relying on serum creatinine (14). CKD was categorized into three groups: (1) No CKD: eGFR ≥ 60 ml/min/1.73 m^2^, ACR < 30 mg/g, and no history of weak/failing kidneys; (2) Stages G1–G3: eGFR ≥ 60 ml/min/1.73 m^2^ and ACR ≥ 30 mg/g or eGFR between 30 and 59 ml/min/1.73 m^2^; (3) Stages G4–G5: eGFR < 30 ml/min/1.73 m^2^ or received dialysis in the past 12 months.

### 2.3 Diabetes

For the assessment of diabetes, we analyzed measurements of glycated hemoglobin (A1c) and fasting plasma glucose (FPG). The diagnosis of diabetes was established based on any of the following criteria: an A1c level ≥ 6.5%, an FPG level > 126 mg/dL, or a formal diagnosis confirmed by a physician (15).

### 2.4 Age groups

Participants in the study were categorized into three age groups (16). These groups were defined as follows: (1) Young, encompassing individuals aged from 20 to < 45 years; (2) Middle Age, including participants aged 45 to < 60 years; (3) Elderly, comprising individuals aged 60 years and above.

### 2.5 Statistical analysis

Continuous variables were denoted as mean with standard deviations, while categorical variables were expressed as numbers with proportions. NHANES examination sample weights were utilized to adjust for the complex survey design. Complex survey analysis techniques were employed to compute 95% confidence intervals accommodating the sampling weights for national representativeness. Multivariable logistic regression models were employed to investigate potential risk factors for CKD. Statistical analyses were conducted in R software, version 4.1.2, with a significance level of α < 0.05.

## 3 Results

### 3.1 Basic demographic findings

As shown in Table 1, over a span of 20 years, BMI rose steadily, reaching 29.52 kg/m^2^ in 2017–2018. A decline in renal function was observed, with mean eGFR decreasing from 110.09 to 94.96 ml/min/1.73 m^2^. ACR values fluctuated, ending at a higher average in the final period. Serum creatinine and A1c levels increased modestly throughout the study. The proportion of participants by gender remained balanced. CKD prevalence was mostly stable, with a small rise in early-stage (G1-G3) CKD. Notably, diabetes prevalence increased significantly from 12.7 to 20.4%.

**Table 1:** The basic characteristic of study participants aged 20 years and over.

**Characteristic**	**Years**									
	**1999–2000**	**2001–2002**	**2003–2004**	**2005–2006**	**2007–2008**	**2009–2010**	**2011–2012**	**2013–2014**	**2015–2016**	**2017–2018**
	***N*** = **4,880**	***N*** = **5,411**	***N*** = **5,041**	***N*** = **4,979**	***N*** = **5,935**	***N*** = **6,218**	***N*** = **5,560**	***N*** = **5,769**	***N*** = **5,719**	***N*** = **5,569**
Age (years)	44.74 ± 16.45	45.1 ± 16.34	46.23 ± 16.64	46.39 ± 16.19	46.26 ± 15.99	46.87 ± 16.34	46.27 ± 16.25	46.82 ± 16.86	48.35 ± 16.76	47.74 ± 17
BMI (kg/m^2^)	27.6 ± 6.01	27.92 ± 6.08	28.24 ± 6.05	28.49 ± 6.83	28.06 ± 5.83	28.51 ± 6.39	28.43 ± 6.16	28.83 ± 7	29.15 ± 6.86	29.52 ± 7.21
eGFR (ml/min/1.73 m^2^)	110.09 ± 21.01	95.22 ± 21.73	94.26 ± 21.23	91.32 ± 20.47	96.18 ± 20.17	95.62 ± 21.03	96.44 ± 20.28	94.22 ± 20.95	95.97 ± 21.21	94.96 ± 21.75
ACR (mg/g)	27.57 ± 289.49	29.27 ± 223.88	21.12 ± 147.54	18.73 ± 79.6	23.31 ± 171.27	21.32 ± 248.4	15.76 ± 71.98	17.96 ± 118.52	21.64 ± 106.62	35.85 ± 285.59
Serum creatinine (mg/dL)	0.73 ± 0.34	0.89 ± 0.28	0.89 ± 0.23	0.92 ± 0.24	0.86 ± 0.24	0.87 ± 0.38	0.86 ± 0.22	0.88 ± 0.24	0.85 ± 0.3	0.87 ± 0.28
A_1c_ (%)	5.37 ± 0.91	5.46 ± 0.81	5.47 ± 0.75	5.33 ± 0.61	5.42 ± 0.45	5.49 ± 0.56	5.45 ± 0.54	5.43 ± 0.54	5.48 ± 0.54	5.67 ± 0.93
FPG (mg/dL)	100.29 ± 30.83	101.78 ± 29.64	100.32 ± 26.75	99.63 ± 19.97	101.14 ± 13.56	100.02 ± 17.57	100.06 ± 15.72	99.8 ± 16.99	103.48 ± 17.79	110.45 ± 32.21
TG (mg/dL)	143.03 ± 103.02	152.62 ± 178.57	148.31 ± 132.14	138.02 ± 108.71	131.58 ± 97.2	125.41 ± 100.07	129.14 ± 101.84	117.21 ± 96.4	110.98 ± 77.62	112.94 ± 98.8
HDL (mg/dL)	49.94 ± 15.07	51.34 ± 15.38	54.13 ± 15.83	55.48 ± 15.94	53.74 ± 15.51	54.67 ± 16.44	53.26 ± 14.49	54.29 ± 16.1	56.85 ± 18.32	54.11 ± 15.6
2HPG (mg/dL)	-	-	-	115.93 ± 51.98	117.39 ± 45.67	116.44 ± 49.82	114.91 ± 47.72	114.17 ± 46.22	118.8 ± 48.22	-
**Gender**										
Male	2,269 (46.5%)	2,536 (46.9%)	2,418 (48%)	2,387 (47.9%)	2,910 (49%)	3,006 (48.3%)	2,740 (49.3%)	2,758 (47.8%)	2,747 (48.0%)	2,702 (48.5%)
Female	2,611 (53.5%)	2,875 (53.1%)	2,623 (52%)	2,592 (52.1%)	3,025 (51%)	3,212 (51.7%)	2,820 (50.7%)	3,011 (52.2%)	2,972 (52.0%)	2,867 (51.5%)
**Age group**										
Young	2,110 (43.24%)	2,408 (44.5%)	2,160 (42.85%)	2,359 (47.38%)	2,381 (40.12%)	2,643 (42.51%)	2,419 (43.51%)	2,503 (43.39%)	2,427 (42.44%)	2,095 (37.62%)
Middle age	936 (19.18%)	1,131 (20.9%)	980 (19.44%)	1,050 (21.09%)	1,400 (23.59%)	1,502 (24.16%)	1,350 (24.28%)	1,425 (24.7%)	1,391 (24.32%)	1,324 (23.77%)
Elderly	1,834 (37.58%)	1,872 (34.6%)	1,901 (37.71%)	1,570 (31.53%)	2,154 (36.29%)	2,073 (33.34%)	1,791 (32.21%)	1,841 (31.91%)	1,901 (33.24%)	2,150 (38.61%)
**CKD stage**										
No CKD	3,349 (68.6%)	3,724 (68.8%)	3,472 (68.9%)	3,481 (69.9%)	4,145 (69.8%)	4,630 (74.5%)	3,906 (70.3%)	4,246 (73.6%)	4,129 (72.2%)	3,827 (68.7%)
Stage G1–G3	624 (12.8%)	835 (15.4%)	827 (16.4%)	833 (16.7%)	974 (16.4%)	896 (14.4%)	826 (14.9%)	905 (15.7%)	846 (14.8%)	921 (16.5%)
Stage G4–G5	30 (0.6%)	57 (1.1%)	57 (1.1%)	54 (1.1%)	62 (1%)	69 (1.1%)	66 (1.2%)	61 (1.1%)	56 (1%)	56 (1%)
Missing	877 (18%)	795 (14.7%)	685 (13.6%)	611 (12.3%)	754 (12.7%)	623 (10%)	762 (13.7%)	557 (9.7%)	688 (12%)	765 (13.7%)
**Race**										
Mexican American	1,282 (26.3%)	1,113 (20.6%)	985 (19.5%)	1,003 (20.1%)	1,033 (17.4%)	1,140 (18.3%)	540 (9.7%)	767 (13.3%)	995 (17.4%)	735 (13.2%)
Other Hispanic	310 (6.4%)	237 (4.4%)	152 (3.0%)	154 (3.1%)	666 (11.2%)	632 (10.2%)	578 (10.4%)	508 (8.8%)	768 (13.4%)	517 (9.3%)
Non-Hispanic White	2,214 (45.4%)	2,858 (52.8%)	2,689 (53.3%)	2,495 (50.1%)	2,761 (46.5%)	2,976 (47.9%)	2,041 (36.7%)	2,472 (42.8%)	1,863 (32.6%)	1,935 (34.7%)
Non-Hispanic Black	910 (18.6%)	1,012 (18.7%)	994 (19.7%)	1,123 (22.6%)	1,227 (20.7%)	1,122 (18.0%)	1,455 (26.2%)	1,177 (20.4%)	1,198 (20.9%)	1,298 (23.3%)
Other Race	164 (3.4%)	191 (3.5%)	221 (4.4%)	204 (4.1%)	248 (4.2%)	348 (5.6%)	946 (17.0%)	845 (14.6%)	895 (15.6%)	1,084 (19.5%)
**Weak/failing kidneys**										
Yes	19 (0.3%)	145 (2.7%)	144 (2.9%)	136 (2.7%)	179 (3%)	158 (2.5%)	200 (3.6%)	187 (3.2%)	239 (4.2%)	223 (4%)
No	204 (3.7%)	5,246 (97%)	4,885 (96.9%)	4,829 (97%)	5,745 (96.8%)	6,047 (97.2%)	5,354 (96.3%)	5,574 (96.6%)	5,472 (95.7%)	5,337 (95.8%)
Missing	5,346 (96%)	20 (0.4%)	12 (0.2%)	14 (0.3%)	11 (0.2%)	13 (0.2%)	6 (0.1%)	8 (0.1%)	8 (0.1%)	9 (0.2%)
**Received dialysis**										
Yes	-	19 (0.4%)	9 (0.2%)	17 (0.3%)	22 (0.4%)	22 (0.4%)	27 (0.5%)	20 (0.3%)	20 (0.3%)	19 (0.3%)
No	-	126 (2.3%)	135 (2.7%)	119 (2.4%)	157 (2.6%)	136 (2.2%)	173 (3.1%)	167 (2.9%)	218 (3.8%)	204 (3.7%)
Missing	-	5,266 (97.3%)	4,897 (97.1%)	4,843 (97.3%)	5,756 (97%)	6,060 (97.5%)	5,360 (96.4%)	5,582 (96.8%)	5,481 (95.8%)	5,346 (96%)
**Diabetes**										
Yes	618 (12.7%)	678 (12.5%)	687 (13.6%)	709 (14.2%)	1,116 (18.8%)	1,074 (17.3%)	999 (18.0%)	986 (17.1%)	1,130 (19.8%)	1,136 (20.4%)
No	4,253 (87.2%)	4,729 (87.4%)	4,346 (86.2%)	4,262 (85.6%)	4,811 (81.1%)	5,138 (82.6%)	4,551 (81.9%)	4,771 (82.7%)	4,577 (80.0%)	4,420 (79.4%)
Missing	9 (0.1%)	4 (0.1%)	8 (0.2%)	8 (0.2%)	8 (0.1%)	6 (0.1%)	10 (0.2%)	12 (0.2%)	12 (0.2%)	13 (0.2%)

### 3.2 The overall prevalence of CKD stages

**Figure 1:**
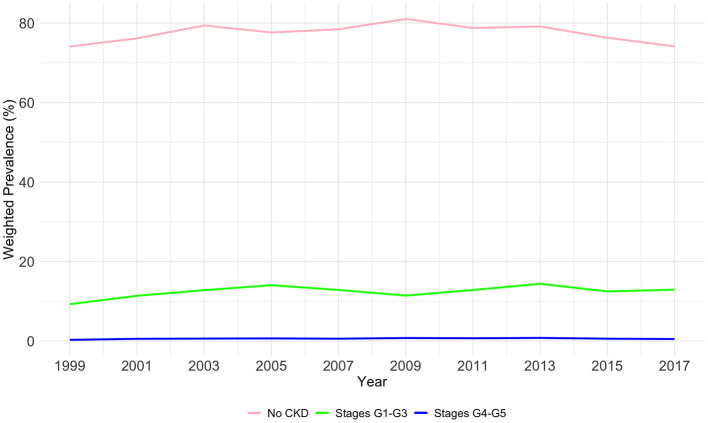
The overall weighted prevalence of CKD stage by years among study population.

As shown in Table 2 and Figure 1, The prevalence of CKD stages among the US population from 1999 to 2018 demonstrates significant variability across stages and years (*P* < 0.01). In the initial phase of the study period (1999–2000), the majority of the study population did not exhibit CKD (74.08%), with 9.28% falling within stages G1–G3, and a mere 0.3% within stages G4–G5. A gradual increase in the prevalence of stages G1–G3 was observed, reaching 14.4% by 2013–2014, before a slight reduction to 12.93% in 2017–2018. Conversely, the percentage of participants with no CKD, after peaking at 81.01% in 2009–2010, showed a decline to 74.12% by the end of the study period. The prevalence of more advanced CKD (stages G4–G5) initially rose from 0.3% in 1999–2000 to 0.79% in 2013–2014, followed by a decrease to 0.51% in 2017–2018.

**Table 2:** The overall prevalence of CKD stages by years among study population (%).

**Years**	**No CKD**	**Stage G1–G3**	**Stage G4–G5**	***P*-value**
1999–2000	74.08 (73.80, 74.40)	9.28 (9.10, 9.45)	0.30 (0.27, 0.33)	< 0.01
2001–2002	76.12 (75.59, 76.38)	11.39 (11.19, 11.58)	0.57 (0.52, 0.62)		
2003–2004	79.38 (79.13, 79.63)	12.80 (12.59, 13.00)	0.63 (0.58, 0.68)		
2005–2006	77.61 (77.35, 77.87)	14.05 (13.83, 14.26)	0.67 (0.62, 0.72)		
2007–2008	78.42 (78.17, 78.67)	12.86 (12.65, 13.07)	0.60 (0.55, 0.65)		
2009–2010	81.01 (80.77, 81.25)	11.45 (11.25, 11.64)	0.77 (0.72, 0.82)		
2011–2012	78.73 (78.48, 78.98)	12.84 (12.63, 13.04)	0.71 (0.66, 0.76)		
2013–2014	79.13 (78.88, 79.38)	14.40 (14.18, 14.62)	0.79 (0.74, 0.84)		
2015–2016	76.28 (76.02, 76.54)	12.49 (12.29, 12.69)	0.60 (0.55, 0.65)		
2017–2018	74.12 (73.84, 74.39)	12.93 (12.72, 13.31)	0.51 (0.47, 0.55)		

### 3.3 Weighted prevalence of CKD stage G4–G5

**Figure 2:**
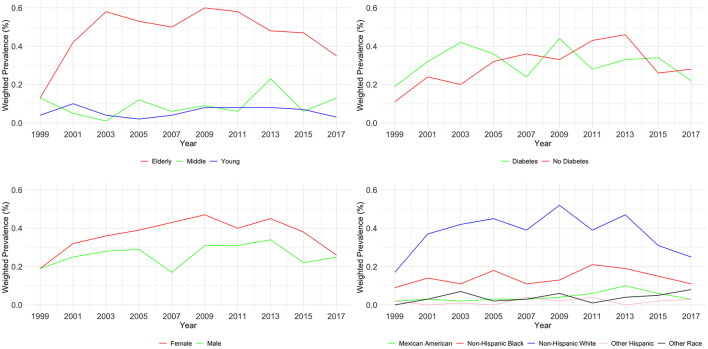
Weighted prevalence of CKD stage G4–G5 across various demographic groups.

As shown in Table 3 and Figure 2, an analysis of CKD stages G4–G5 from 1999 to 2018 across demographic groups shows the prevalence is relatively stable across age and diabetes status, with no significant changes (*P* = 0.124 and *P* = 0.236, respectively). Age-wise, the prevalence in the young remained low, the middle-aged group peaked at 0.23% in 2013–2014, and the elderly peaked at 0.6% in 2009–2010. For diabetes, the highest prevalence was 0.46% in 2013–2014 for diabetics. Gender differences were not significant (*P* = 0.873), with both male and female groups showing minor fluctuations over the years. Significant changes over time were noted for races (*P* < 0.01), with Mexican Americans, non-Hispanic White people, and Non-Hispanic Black people showing peaks at various points, but all groups ended with a decreased prevalence in 2017–2018.

**Table 3:** Weighted prevalence of CKD stage G4–G5 across various demographic groups (%).

	**Years**										***P*–value**
	**1999–2000**	**2001–2002**	**2003–2004**	**2005–2006**	**2007–2008**	**2009–2010**	**2011–2012**	**2013–2014**	**2015–2016**	**2017–2018**	
**Age group**											
Young ( ≤ 44)	0.04 (0.03, 0.05)	0.10 (0.10, 0.11)	0.04 (0.03, 0.05)	0.02 (0.01, 0.03)	0.04 (0.03, 0.05)	0.08 (0.06, 0.09)	0.08 (0.06, 0.09)	0.08 (0.06, 0.09)	0.07 (0.05, 0.09)	0.03 (0.02, 0.04)	0.124
Middle age (45–59)	0.13 (0.11, 0.15)	0.05 (0.04, 0.06)	0.01 (0.004, 0.02)	0.12 (0.10, 0.14)	0.06 (0.04, 0.08)	0.09 (0.07, 0.11)	0.06 (0.04, 0.08)	0.23 (0.20, 0.26)	0.06 (0.04, 0.08)	0.13 (0.11, 0.15)	
Elderly (≥60)	0.13 (0.11, 0.15)	0.42 (0.38, 0.46)	0.58 (0.53, 0.63)	0.53 (0.48, 0.58)	0.50 (0.46, 0.54)	0.60 (0.55, 0.65)	0.58 (0.53, 0.63)	0.48 (0.44, 0.52)	0.47 (0.43, 0.51)	0.35 (0.31, 0.39)	
**Diabetes**											
No	0.11 (0.09, 0.13)	0.24 (0.21, 0.27)	0.20 (0.17, 0.23)	0.32 (0.28, 0.36)	0.36 (0.32, 0.40)	0.33 (0.29, 0.37)	0.43 (0.39, 0.47)	0.46 (0.42, 0.50)	0.26 (0.23, 0.29)	0.28 (0.25, 0.31)	0.236
Yes	0.19 (0.16, 0.22)	0.32 (0.28, 0.36)	0.42 (0.38, 0.46)	0.36 (0.32, 0.40)	0.24 (0.21, 0.27)	0.44 (0.40, 0.48)	0.28 (0.25, 0.31)	0.33 (0.29, 0.37)	0.34 (0.30, 0.385)	0.22 (0.19, 0.25)	
**Gender**											
Male	0.19 (0.16, 0.22)	0.25 (0.22, 0.28)	0.28 (0.25, 0.31)	0.29 (0.26, 0.32)	0.17 (0.14, 0.20)	0.31 (0.28, 0.34)	0.31 (0.28, 0.34)	0.34 (0.30, 0.38)	0.22 (0.19, 0.25)	0.22 (0.22, 0.28)	0.873
Female	0.11 (0.09, 0.13)	0.32 (0.28, 0.36)	0.36 (0.32, 0.40)	0.39 (0.35, 0.43)	0.43 (0.36, 0.44)	0.47 (0.43, 0.51)	0.40 (0.36, 0.44)	0.45 (0.41, 0.49)	0.38 (0.34, 0.42)	0.26 (0.13, 0.29)	
**Race**											
Mexican American	0.02 (0.01, 0.03)	0.03 (0.02, 0.04)	0.02 (0.01, 0.03)	0.03 (0.02, 0.04)	0.03 (0.02, 0.04)	0.04 (0.03, 0.05)	0.06 (0.04,0.08)	0.10 (0.08, 0.12)	0.06 (0.04, 0.08)	0.03 (0.02, 0.04)	< 0.01
Other Hispanic	0.02 (0.01, 0.03)	0.00 (0.00,0.00)	0.01 (0.004, 0.02)	0.00 (0.00,0.00)	0.04 (0.03, 0.05)	0.02 (0.01, 0.03)	0.04 (0.03, 0.05)	0.00 (0.00,0.00)	0.02 (0.01, 0.03)	0.03 (0.02, 0.04)	
Non-Hispanic White	0.17 (0.14, 0.20)	0.37 (0.33, 0.41)	0.42 (0.38, 0.46)	0.45 (0.41, 0.49)	0.39 (0.35, 0.43)	0.52 (0.48, 0.56)	0.39 (0.35, 0.43)	0.47 (0.43, 0.51)	0.31 (0.28, 0.34)	0.25 (0.22, 0.28)	
Non-Hispanic Black	0.09 (0.07, 0.11)	0.14 (0.12, 0.16)	0.11 (0.09, 0.13)	0.18 (0.15, 0.21)	0.11 (0.09, 0.13)	0.13 (0.11, 0.15)	0.21 (0.18, 0.24)	0.19 (0.16, 0.22)	0.15 (0.13, 0.17)	0.11 (0.09, 0.13)	
Other Race	0.00 (0.00, 0.00)	0.03 (0.02,0.04)	0.07 (0.05, 0.09)	0.02 (0.01, 0.03)	0.03 (0.02, 0.04)	0.06 (0.04, 0.08)	0.01 (0.004, 0.02)	0.04 (0.03, 0.05)	0.05 (0.04, 0.06)	0.08 (0.06, 0.10)	

### 3.4 Weighted prevalence of CKD stage G1–G3

**Figure 3:**
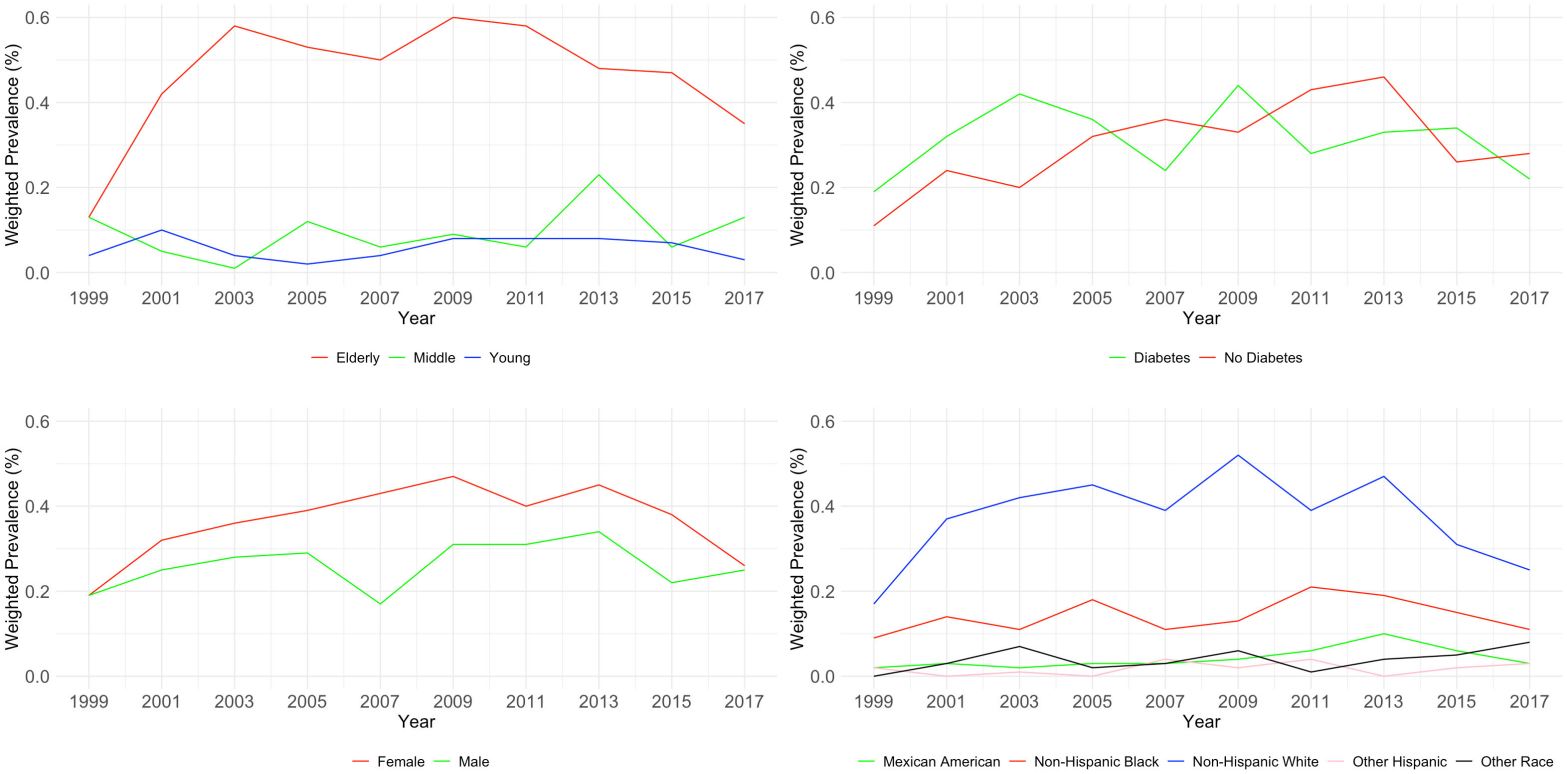
Weighted prevalence of CKD stage G1–G3 across various demographic groups.

As shown in Table 4 and Figure 3, the examination of CKD stages G1–G3 from 1999 to 2018 revealed significant variations across age, diabetes status, and race (all *P* < 0.01), but not gender (*P* = 0.739). Young adults (20–44) showed an initial prevalence of 3.16%, which varied slightly, ending at 2.33%. Middle-aged individuals (45–59) started at 1.78% and had an incremental rise to 2.94%. The elderly (60–89) had a higher prevalence, beginning at 4.34% and reaching 7.66% by 2018.

**Table 4:** Weighted prevalence of CKD stage G1–G3 across various demographic groups (%).

	**Years**										***P*–value**
	**1999–2000**	**2001–2002**	**2003–2004**	**2005–2006**	**2007–2008**	**2009–2010**	**2011–2012**	**2013–2014**	**2015–2016**	**2017–2018**	
**Age group**											
Young (20–44)	3.16 (305, 3.27)	2.47 (2.37, 2.57)	2.45 (2.35, 2.55)	2.82 (2.72, 2.92)	2.78 (2.68, 2.88)	2.02 (1.93, 2.11)	2.49 (2.39, 2.59)	3.26 (3.15, 3.37)	2.29 (2.20, 2.38)	2.33 (2.24, 2.42)	< 0.01
Middle age (45–59)	1.78 (1.70, 1.86)	2.77 (2.67,2.87)	2.71 (2.61, 2.81)	3.04 (2.93, 3.15)	2.90 (2.80,3.00)	2.15 (2.06,2.24)	2.96 (2.85,3.07)	3.07 (2.96, 3.18)	2.49 (2.39,2.59)	2.94 (2.84,3.04)	
Elderly (60–89)	4.34 (4.21, 4.47)	6.15 (6.00, 6.30)	7.64 (7.48, 7.80)	8.20 (8.03, 8.37)	7.17 (7.01, 7.33)	7.29 (7.13, 7.45)	7.39 (7.23, 7.55)	8.07 (7.90, 8.24)	7.71 (7.54, 7.88)	7.66 (7.50, 7.82)	
**Diabetes**											
No	2.01 (1.92, 2.09)	3.17 (3.06, 3.28)	3.76 (3.64, 3.88)	4.07 (3.95, 4.19)	4.20 (4.08, 4.32)	4.01 (3.89, 4.13)	4.25 (4.12, 4.38)	4.49 (4.36, 4.62)	4.39 (4.26, 4.52)	4.89 (4.76, 5.02)	< 0.01
Yes	7.27 (7.11, 7.43)	8.22 (8.05, 8.39)	9.03 (8.85, 9.21)	9.98 (9.79, 10.17)	8.65 (8.48, 8.82)	7.45 (7.29, 7.61)	8.59 (8.42, 8.76)	9.91 (9.72, 10.10)	8.10 (7.93, 8.27)	8.05 (7.88, 8.22)	
**Gender**											
Male	3.94 (3.82, 4.06)	5.02 (4.88,5.16)	5.67 (5.53,5.81)	5.77 (5.63, 5.91)	5.21 (5.07, 5.35)	5.04 (4.90, 5.18)	5.62 (5.48, 5.76)	5.95 (5.80, 6.10)	5.33 (5.19, 5.47)	5.78 (5.64, 5.92)	0.739
Female	5.34 (5.20, 5.48)	6.37 (6.22, 6.52)	7.13 (6.97, 7.29)	8.28 (8.11, 8.45)	7.65 (7.49, 7.81)	6.41 (6.26, 6.56)	7.22 (7.06, 7.38)	8.44 (8.27, 8.61)	7.16 (7.00, 7.32)	7.15 (6.99, 7.31)	
**Race**											
Mexican American	0.54 (0.49, 0.59)	0.65 (0.60, 0.70)	0.81 (0.75, 0.87)	0.85 (0.79, 0.91)	0.94 (0.88, 1.00)	0.90 (0.94, 0.96)	0.87 (0.81, 0.93)	1.02 (0.96, 1.08)	0.97 (0.91, 1.03)	0.95 (0.89, 1.01)	< 0.01
Other Hispanic	0.89 (0.83, 0.95)	0.67 (0.62, 0.72)	0.43 (0.39, 0.47)	0.50 (0.46, 0.54)	0.57 (0.52, 0.62)	0.46 (0.42, 0.50)	0.74 (0.69, 0.79)	0.69 (0.64, 0.74)	0.54 (0.49, 0.59)	0.74 (0.69, 0.79)	
Non-Hispanic White	6.14 (5.99, 6.29)	494 (8.39%)	484 (9.16%)	471 (10.4%)	519 (9.37%)	466 (8.12%)	337 (8.64%)	455 (10.22%)	341 (8.52%)	391 (8.45%)	
Non-Hispanic Black	1.11 (1.04, 1.17)	1.28 (1.21, 1.35)	1.42 (1.35, 1.49)	1.66 (1.58, 1.74)	1.41 (1.34, 1.48)	1.37 (1.30, 1.44)	1.66 (1.58, 1.74)	1.57 (1.49, 1.65)	1.43 (1.36, 1.50)	1.50 (1.42, 1.58)	
Other Race	0.61 (0.56, 0.66)	0.39 (0.35,0.43)	0.97 (0.91, 1.03)	0.63 (0.58, 0.68)	0.56 (0.51, 0.61)	0.60 (0.55, 0.65)	0.93 (0.87, 0.99)	0.89 (0.83, 0.95)	1.02 (0.96, 1.08)	1.29 (1.22, 1.36)	

For individuals with diabetes, there was a notable increase from 2.01 to 4.89%, while those without diabetes started at 7.27% and slightly increased to 8.05%. Gender-related prevalence was stable, with males starting at 3.94% and ending at 5.78%, and females from 5.34 to 7.15%. Racial disparities were evident. Mexican Americans increased from 0.54 to 0.95%, Other Hispanics fluctuated, ending at 0.74%. Non-Hispanic White people and Black people showed a rise, with White people starting at 6.14 and ending at 8.45%, and Black people from 1.11 to 1.50%. Other Races had an increase from 0.61 to 1.29%. Multivariable logistic regression analysis found evidence to support common risk factors for CKD as shown in Table 5.

**Table 5:** Potential risk factors associated with CKD in NHANES.

**Variables**	**OR (95% CI)**
Age	1.25 (1.10, 1.35)
Gender	1.36 (1.15, 1.56)
Education	0.95 (0.70, 1.35)
Current Smoking	1.50 (1.23, 1.96)
Obesity	1.87 (1.45, 2.39)
Diabetes	2.40 (2.11, 2.65)
Hypertension	2.20 (1.89, 2.59)
Dyslipidemia	2.54 (2.10, 2.90)

## 4 Discussion

Our study offers a comprehensive analysis of CKD prevalence trends in the United States over a span of nearly two decades, from 1999 to 2018, utilizing data from NHANES. Our findings not only corroborate but also expand upon the existing body of literature, uncovering nuanced trends and disparities across various demographic segments. In this study, our analyses indicate that early CKD stages (G1–G3) increased from 9.28% in 1999–2000 to 12.93% in 2017–2018, while advanced CKD stages (G4–G5) showed a slight increase in prevalence from 0.3% in 1999–2000 to 0.51% in 2017–2018. The prevalence we observed is similar to a systematic review focusing on CKD in Asia, and the similar prevalence between the developed country such as the USA, and the developing country such as China is also consistent with a review (17, 18). Figures 2, 3 in our study delineate the potential impact of various risk factors on CKD prevalence, as deduced from our analysis. These risk factors are pivotal in shaping public health strategies aimed at the prevention and management of CKD.

### 4.1 Temporal trends in CKD prevalence

Our observations reveal dynamic shifts in CKD prevalence, particularly noticeable in the early to moderate stages (G1–G3). Recent studies utilizing NHANES data have provided valuable insights into the temporal trends of CKD prevalence. Castro and Coresh (19) observed an increase in the prevalence of early-stage CKD between 1988–1994 and 1999–2004, indicating a growing burden of the disease in its initial stages. However, Murphy et al. (20) reported a stabilization in the prevalence of stage 3 and 4 CKD since the early 2000's, suggesting effective management and awareness of CKD risk factors. Foley (21) highlighted the impact of measurement methods on CKD prevalence trends, noting differences in results based on serum creatinine and cystatin C levels. Additionally, Hsu and Powe (11) observed a plateau in the prevalence of diabetic kidney disease since the mid-2000's, reflecting advancements in managing diabetes-related kidney complications. Wu et al. (22) further emphasized the consistent prevalence of CKD among patients with Type 2 Diabetes Mellitus (T2DM), particularly in older adults and certain ethnic groups, underscoring the need for targeted interventions in these populations. Intriguingly, the prevalence rates for the more severe stages of CKD (G4–G5) displayed a remarkable consistency throughout the study period. This stability might be attributed to the effectiveness of medical interventions at these stages or the success of timely medical care in preventing the progression from early to advanced stages of CKD (20).

### 4.2 Age-related disparities

Our analysis indicated that both early and advanced stages of CKD were more prevalent in the elderly population, reaffirming age as a significant risk factor for CKD. This trend is consistent with the gradual increase in the mean age of NHANES participants over the years, which could partially account for the observed escalation in CKD prevalence rates. A study by Malekmakan et al. (23) in Southern Iran found that the prevalence of CKD stages III–V increased significantly with age, particularly in individuals aged 80 years and above, and identified female gender as a strong risk factor for CKD in the elderly. Additionally, Liu et al. (24) conducted a retrospective analysis in elderly Chinese patients and found that estimated glomerular filtration rate (eGFR) values decreased with age, while the incidence of reduced renal function and other urinary abnormalities increased with age. This study highlights the importance of monitoring and managing renal function in the elderly, considering the increased risk of CKD with advancing age (24). These studies collectively underscore the importance of understanding age-related disparities in CKD prevalence and progression. They highlight the need for targeted interventions and management strategies that consider age, sex, and other demographic factors to effectively address the growing burden of CKD in the elderly population.

### 4.3 Diabetes and CKD

A significant correlation was found between diabetes mellitus and CKD, particularly in the advanced stages. This association aligns with the existing literature that identifies diabetes as a major contributor to the onset and progression of CKD (22). Our study adds to the growing body of evidence underscoring the critical interplay between diabetes and CKD. Recent systematic reviews and studies have reinforced the understanding that diabetes, often in conjunction with hypertension, plays a pivotal role in the progression of CKD. Research by Saldivi highlights the positive association between diabetes and the progression of CKD, emphasizing the need for further exploration into the mechanisms linking these conditions (25). Additionally, studies like those conducted by Guo et al. (26) and Ren et al. (27) have expanded our understanding of the relationship between diabetes and CKD. They reveal how factors such as diet-induced inflammation and cardiovascular risk are intricately connected to CKD prevalence and progression in diabetic patients. Furthermore, Dattani et al. (28) provide insights into the association of CKD stage 3a with significant multi-morbidity in diabetic patients, particularly noting the role of albuminuria levels. These findings collectively highlight the significant role of diabetes in the development and progression of CKD. They underscore the need for integrated management strategies that address both diabetes and CKD, considering factors such as hypertension, dietary inflammation, cardiovascular risk, and albuminuria status. This integrated approach is crucial for developing effective interventions and management plans for patients suffering from both diabetes and CKD.

### 4.4 Gender and racial disparities

While the differences in CKD prevalence based on gender were relatively modest, racial disparities were more pronounced. Our study found that Non-Hispanic White people and Non-Hispanic Black people exhibited higher CKD prevalence rates compared to other racial groups, corroborating findings from previous research (29). This underscores the necessity for targeted healthcare interventions and policies that address these disparities, ensuring equitable healthcare access and management for all racial groups. Previous studies have shown differences in CKD levels across socioeconomic levels, and that different lifestyles, access to healthcare, and malnutrition may account for this phenomenon. Individuals living in low-income countries lack access to kidney disease diagnosis, prevention, or treatment, and most of them with CKD are unaware that they have this condition and therefore do not seek treatment (30). In addition, maternal factors such as malnutrition and poor health literacy can attribute to the adverse uterine environment which causes CKD risk factors including small for gestational age, low birthweight, and prematurity and further leads to CKD in later life (31). These phenomena combined with aging population and population growth will translate to a large increase in the prevalence of CKD in low-income countries in the coming decades (32). Yinusa et al. (33) emphasized the need for immediate intervention to improve healthcare conditions for minorities experiencing CKD. Their research used system dynamics modeling to illustrate the relationships among dynamic factors influencing the incidence and prevalence of CKD, highlighting inherent challenges in the treatment and management of this disease due to healthcare disparities (33).

Recent studies have further elucidated these disparities. For instance, McCormick et al. (34) investigated racial differences in gout prevalence, a condition closely associated with CKD, and found significant disparities between Black people and White individuals in the US. The study suggested that these disparities could be explained by factors such as diet, social determinants of health, and CKD itself (34). Additionally, Prince et al. (42) conducted a population-based retrospective study focusing on HIV mortality in the United States, which revealed significant racial and gender disparities. Although not directly related to CKD, this study provides insights into the broader context of health disparities that can inform targeted interventions in CKD management (34). Furthermore, Shahid et al. (43) explored gender disparities in low-density lipoprotein cholesterol management across various cardiovascular diseases, providing a perspective on how these disparities might also manifest in CKD management (35). These studies collectively highlight the significant role of both gender and racial disparities in the prevalence and management of CKD. They underscore the need for healthcare systems to recognize and address these disparities through targeted interventions, ensuring equitable healthcare access and outcomes for all individuals, regardless of their race or gender.

### 4.5 Clinical implications

The findings of our study carry substantial clinical implications for the early detection and management of CKD. In this context, primary care physicians are pivotal, often serving as the initial point of contact for patients. Enhancing awareness and providing additional training for these healthcare professionals about CKD's risk factors and early symptoms can lead to more prompt diagnoses and timely interventions. A patient-centered self-management (PCSM) approach, as identified by Lin and Hwang (36), is crucial in CKD management. This approach integrates health literacy and information technology interventions, emphasizing the importance of unified patient education in addressing the challenges of CKD management. Implementing a standardized and universal integrated PCSM model could significantly improve treatment outcomes in CKD patients (36). Moreover, the role of telemedicine and interdisciplinary teams in managing CKD-related complications, such as difficult-to-control hypertension, has been highlighted by Dopp et al. (37). Their study demonstrates the effectiveness of a collaborative nephrologist–pharmacist telehealth clinic, suggesting that virtual care models can be a potent tool in CKD management (37). Additionally, mobile health interventions, as shown in the SINEMA trial by Yan et al. (38), have proven effective in managing non-communicable diseases through primary healthcare. This underscores the potential of similar mobile health strategies in improving CKD management, especially in rural or underserved areas (38). Furthermore, the importance of virtual education pathways in managing chronic conditions is evident in the work of Soliman (39), who found significant improvements in glycemic control through a virtual diabetes self-management education pathway. This indicates the potential for similar virtual interventions in enhancing CKD management (39).

Patient education in lifestyle modifications remains crucial. Educating patients about dietary changes, increasing physical activity, and effective weight management could play a crucial role in preventing the onset or progression of CKD. Considering the strong link between CKD and comorbid conditions such as diabetes and hypertension, integrated care models that address these conditions concurrently could prove more effective. The observed disparities in CKD prevalence among different demographic groups call for a more personalized approach to CKD management, taking into account factors such as age, race, and existing comorbidities. Tailoring interventions to meet the specific needs of individual patients can enhance the effectiveness of CKD management strategies. Advancements in technology, such as telemedicine and mobile health applications, present new avenues for CKD monitoring and management. These technological solutions can facilitate better patient engagement, continuous monitoring, and more efficient management of CKD, thereby improving patient outcomes and quality of life.

### 4.6 Public health significance

The results of our study have profound implications for public health in the United States, particularly in the context of CKD. CKD, as a chronic health condition, not only impacts the individuals directly affected but also places a considerable strain on the healthcare system. The observed increase in the prevalence of early-stage CKD in our study suggests a looming escalation in the future burden of advanced kidney diseases, including ESRD. The progression to ESRD is associated with significant healthcare costs, largely due to the requirements for dialysis and kidney transplantation (40).

Additionally, CKD is recognized as a risk multiplier for cardiovascular diseases, thereby amplifying its public health importance. As CKD advances, the risk of cardiovascular events and associated mortality intensifies, highlighting the urgent need for early intervention and effective management of CKD to mitigate cardiovascular risks (41). The disparities in CKD prevalence among different demographic groups, such as by age, race, and diabetes status, underscore the necessity for tailored public health strategies. These strategies should prioritize targeted screening, early detection, and the development of culturally sensitive management plans, particularly for populations at higher risk.

Our study also emphasizes the critical role of addressing modifiable risk factors for CKD, including hypertension and diabetes, through comprehensive public health initiatives. Effective control and management of these conditions can play a significant role in reducing the incidence of CKD or decelerating its progression. The increasing prevalence of CKD in the United States, as evidenced by our research, necessitates immediate and concerted public health actions. These actions should focus on enhancing awareness about CKD, implementing robust prevention strategies, and improving access to healthcare services. Such measures are essential for the early detection and management of CKD, ultimately aiming to reduce the overall burden of this disease on individuals and the healthcare system.

### 4.7 Limitations and future directions

While our study provides valuable insights into CKD prevalence trends in the United States, it is important to acknowledge its limitations and the directions for future research. One of the primary limitations of our study is its cross-sectional design, which constrains our ability to establish a causal relationship between various risk factors and the prevalence of CKD. To gain a more comprehensive and robust understanding, future research endeavors should employ longitudinal study designs. Such designs would allow for the observation of changes and trends over time, offering a clearer picture of the progression and dynamics of CKD. In addition, although recognized measurement methods were employed, CKD status was classified solely based on serum creatinine and ACR, potentially introducing misclassification bias (20). However, this probably did not bias our assessment of temporal trends because misclassification is likely to be non-differential concerning calendar year (20). Furthermore, using data from NHANES, our analysis only focused on people living in the US, which limits the generalizability to individuals living in low-income countries.

Moreover, our study did not encompass all potential risk factors that might influence CKD prevalence. For instance, factors like blood lead levels, which have been suggested to have an association with CKD (19), were not included in our analysis. This indicates the need for future studies to incorporate a broader range of risk factors to fully understand the multifaceted nature of CKD. Given the evolving landscape of CKD prevalence and its interplay with various risk factors, future research should prioritize longitudinal studies. These studies would be instrumental in elucidating the progression of CKD and its long-term implications on public health. They could also shed light on the effectiveness of current intervention strategies and pinpoint areas where novel approaches are necessary.

Additionally, there is a significant opportunity for research focusing on the genetic underpinnings of CKD, particularly in populations that are at higher risk. Such research could uncover new insights into the disease mechanisms and identify potential therapeutic targets. Exploring the role of environmental factors, lifestyle modifications, and dietary habits in the development and progression of CKD is also a promising avenue. These aspects are crucial in understanding the full spectrum of factors contributing to CKD. Finally, assessing the impact of healthcare policies and access to care on the prevalence of CKD is vital. This line of inquiry can inform more effective public health strategies and promote equitable healthcare provision. Understanding how different healthcare systems and policies influence CKD prevalence and management can lead to more tailored and effective approaches in combating this public health challenge.

## 5 Conclusion

Our study offers a detailed and comprehensive analysis of CKD prevalence trends in the United States, uncovering significant disparities across various demographics, including age, diabetes status, and race. These findings carry substantial implications for healthcare planning and the implementation of targeted interventions. The rising prevalence of CKD in the United States, as demonstrated by our research, underscores an urgent need for comprehensive and multifaceted healthcare strategies. These strategies should extend beyond mere treatment of CKD to encompass prevention efforts. There is a critical need to enhance public awareness about CKD risk factors, such as hypertension, diabetes, and obesity, and to advocate for healthier lifestyle choices to mitigate these risks.

Furthermore, the pronounced disparities in CKD prevalence among different demographic groups necessitate targeted interventions. Healthcare policies must be inclusive and equitable, ensuring that high-risk groups, including the elderly, racial minorities, and individuals with comorbid conditions, have access to adequate screening and early intervention services. Our study also highlights the pivotal role of primary care in the management of CKD. Integrating CKD management into primary care practices can lead to improved early detection and intervention, potentially reducing the progression to more advanced stages of CKD and the associated healthcare costs.

In conclusion, the insights gained from our analysis of CKD prevalence trends are invaluable for public health officials, policymakers, and healthcare providers. They underscore the necessity for ongoing research and targeted intervention efforts to effectively address this escalating public health challenge. Our findings serve as a call to action for the development of more effective strategies to combat the growing burden of CKD in the United States.

## Data Availability

The original contributions presented in the study are included in the article/supplementary material, further inquiries can be directed to the corresponding authors.
